# Hepatitis C virus and HIV transmission: parenteral (including percutaneous) routes are still relevant and must receive due attention

**DOI:** 10.3389/fpubh.2026.1790641

**Published:** 2026-05-04

**Authors:** Sandro Vento, Francesca Cainelli

**Affiliations:** 1 Department of Clinical Disciplines, Manash Kozybayev North Kazakhstan University, Petropavlovsk, Kazakhstan; 2 Faculty of Medicine, University of Puthisastra, Phnom Penh, Cambodia; 3 Emirates Hospital Group, Dubai, United Arab Emirates

**Keywords:** blood-borne infections, healthcare-associated infections, hepatitis C virus, HIV, parenteral transmission

## Introduction

Hepatitis C virus (HCV) and HIV share parenteral (including percutaneous) routes of transmission. Even though these routes were considered in the past, attention, as far as HIV is concerned, has subsequently concentrated on sexual routes and vertical transmission.

A case control study done in South Africa in 2016–2020 and recently published served as a reminder of the importance of percutaneous transmission. One thousand one hundred sixty-three men were screened for HIV 1/2, HBsAg, and anti-HCV antibodies and administered a comprehensive questionnaire examining risk factors for potential HCV, HBV, and HIV infection (1). Seroprevalence for HIV was 17.2%, for HBV 6.9%, and for HCV 0.4%. HIV seropositivity was significantly more likely in those who regularly bled during clean-shave haircuts [OR 2.51 (95% CI: 1.16–5.42), *p* = 0.02], had a tattoo/piercing informally done [OR 1.84 (95% CI: 1.13–2.22), *p* = 0.01] or reported sexually transmitted genital lesions or discharge [OR 1.58 (95% CI: 1.13–2.22), *p* = 0.01] (1). HIV/HCV co-infection was more likely in men with the same risk factors. Interestingly, HIV seroprevalence was elevated despite a high circumcision rate (91.2%) (1). In addition, in the state of Jharkhand, eastern India, five thalassemic children have seemingly been found HIV-positive in 2025 following transfusions received at a government hospital (2).

A huge study done in another African country in 2019 is relevant to the issue of HCV and HIV transmission routes. In research involving 156,499 participants in Rwanda, 2.8% were found to be anti-HCV positive and 0.15% had HCV/HIV co-infection (3). History of surgical operation, scarification and exposure to traditional operational practices were associated with both infections, whereas a history of surgery or transfusion was associated with higher probability of HIV infection [OR 1.42 (95% CI: 1.21 to 1.66) and OR 1.48 (1.29 to 1.70), respectively]; a history of physical traumatic assault was associated with a higher probability of HIV infection [OR 1.69 (95% CI: 1.51 to 1.88)] (3).

The above data highlights the importance of percutaneous, healthcare-related and transfusion-related transmissions (that contribute to the impact that HIV and HCV infections have on healthcare systems and to the associated costs) (4, 5) and shows the need for population screening for blood borne viruses, particularly in countries where their burden is considerable.

## Hepatitis C Virus transmission routes

In a study done in 1997 in a rural Egyptian village, with a large sample size (almost 4,000 people) and a 1 in 4 prevalence of anti-HCV, circumcision by informal health care providers was associated with anti-HCV positivity (OR = 1.7, 1.0–3.0) and receiving injections from informal health care providers was associated with 20% higher odds of anti-HCV (*p* = 0.046) (6). A history of hospital admission was also associated with anti-HCV (*p* < 0.001), as were blood transfusions (*p* = 0.028), history of invasive hospital procedures (*p* = 0.004), cesarean section or abortions (*p* = 0.033), surgery, intravenous or urinary catheterization (6). Dental procedures or shaving by community barbers were not related to anti-HCV (6). In partial contrast with the above results, in a study published in 1997 and done in a small town in southern Italy where 170 of 1,352 subjects were anti-HCV positive, the use of glass syringes, and dental therapy were found to be independent predictors of anti-HCV positivity (7). In a 1998 study done in the nine major hospitals of Rawalpindi-Islamabad (Pakistan), anti-HCV antibody-positive cases were more likely to have received injections in the past 10 years (1–10 vs. 0 injections, adjusted OR = 2.8, 95% CI: 1.1–7.1; >10 vs. 0 therapeutic injections, adjusted OR = 3.1, 95% CI: 1.2–7.9) and significantly more likely to have daily face (adjusted OR = 5.1, 95% CI: 1.5–17.0) and armpit shaves (adjusted OR = 2.9, 95% CI: 1.3–6.5) by a barber (8).

Another Egyptian study published in 2004 showed that blood transfusions, invasive procedures (surgery and endoscopy), and shaving at community barbers were associated with anti-HCV positivity (9).

In a Pakistani research published in 2008, 61.45% of HCV infections were likely due to multiple use of needles/syringes, 10.62% to major/minor surgery/dental procedures, 4.26% to blood transfusion and blood products, 3.90% to sharing razors during shaving or circumcision by barbers, piercing instruments, nail clippers, toothbrushes (10). A study in volunteer blood donors in the Northwest Frontier Province of Pakistan, published in the same year, showed that positive donors were more likely to have had 1–2 injections or >2 injections in the past year, or 1–5 intravenous drips or >5 i.v. drips in the past 5 years, and to have been shaved (facial and armpit) by barbers (11).

In an investigation related to the consequences of the reuse of needles and syringes from 2008 to 2015 at a clinic in the Republic of Korea, a distinct HCV genotype 1a was found in 45 patients and in environmental specimens, showing a healthcare-associated outbreak caused by reuse of syringes and contaminated multi-dose vials (12). In Taiwan, a nosocomial HCV outbreak occurred in May-July 2017 in a 30-bed respiratory care ward, where four ventilator-dependent patients acquired HCV infection, and their HCV-RNA sequences were highly similar to those of a patient in the same ward with chronic hepatitis C. The cases had received many more injections than the patients who had remained uninfected; infection control assessment revealed inadequate disinfection of equipment and environment (13). Patient-to-patient transmission most likely was a consequence of contamination during the preparation and administration of injections (13).

In Italy, data from the national surveillance system for acute viral hepatitis for the period 2000–2021 were used to investigate the association between potential risk factors and the risk of acute HBV and HCV infections. The strongest associations for HCV infection were with neurosurgery (OR 11.88; 95% CI: 2.40–58.85), otorhinolaryngological surgery (OR 11.54; 95% CI: 2.55–52.24), vascular surgery (OR 9.52; 95% CI: 3.25–27.87) and ophthalmological surgery (OR 8.32; 95% CI: 2.24–30.92) (14). Endoscopic procedures and/or biopsies were also significantly associated with HCV (OR 3.84; 95% CI: 2.47–5.95) (14).

A 2022 systematic review found a >200% elevated risk for HCV infection among tattooed people (15). Using data from the Cancer Risk Associated with the Body Art of Tattooing (CRABAT) study embedded in the French cohort Constances, French authors examined associations of tattooing with history of HBV and/or HCV infections (self-reported and/or hospital-records). For HCV, the strongest risks were associated with tattooing outside tattoo studios [HR: 4.14 (2.33; 7.35)] (16).

Finally, in a 2025 systematic review of risk factors associated with HCV seroprevalence among the general population in sub-Saharan Africa, higher odds of seroprevalence were observed in people having a history of blood transfusion (OR = 1.81, 95% CI: 1.33–2.45), hospitalization (OR = 1.55, 95% CI: 1.22–1.96), a family history of HCV (OR = 1.52, 95% CI: 1.17–1.96), and having had scarification (OR = 1.29, 95% CI: 1.01–1.64), or a medical operation (OR = 1.28, 95% CI: 1.01–1.62) (17).

Importantly, healthcare-associated transmission of hepatitis C virus continues to occur in community and hospital settings not only in low- and middle-income but also in high-income countries (such as those in European Union and European Economic Area, and the United Kingdom) (18).

## HIV and circumcision

In Kumalo et al.'s (1) study, circumcised men were apparently more likely to have HIV in univariate analysis [OR 2.02 (95% CI: 1.03–3.94), *p* = 0.04], or HIV/HCV [OR 2.04 (1.04–4.00), *p* = 0.04]. In multivariate analysis, the ORs were 1.68 (0.84–3.37; *p* = 0.14), and 1.69 (0.84–3.39; *p* = 0.14), respectively, so the results were not statistically significant (1). Traditional circumcision was done in 950/1,055 (90.05%) cases and medical circumcision in 105/1,055 (9.95%) (1).

It may be that traditional circumcision was done in late adolescence in settings without proper sterilization (19), and that HIV was acquired in that circumstance; however, if that were the case, it is surprising that AIDS had not manifested by the time these people were tested (median age 33.4 years, IQR 27.8–42.0).

It may also be that those circumcised men in this study, believing that they were protected against HIV, have had unprotected sex more frequently or with more partners. In fact, in some communities, traditionally circumcised men have multiple sexual intercourses without using condoms and believe that circumcision completely protect them from HIV acquisition (20).

Anyway, circumcision did not influence HIV acquisition later in life in the study population (1). The authors stated that the lack of a positive effect of circumcision “does suggest that other factors override its potential value in HIV prevention in this group” (1). Indeed, their surprising results point to a greater importance of non-sexual HIV transmission routes in respect of sexual transmission.

## Parenteral HIV transmission

A case-control study was conducted from September through December 2018 in recently identified anti-HIV positive individuals and in seronegative subjects in the district of Unnao, Uttar Pradesh, India (21). In multivariate logistic regression, unsafe injection exposure with already used needles (OR 6.61, 95% CI: 1.80–24.18) and receipt of intramuscular injection in the preceding 5 years (OR 7.20, 95% CI: 1.48–34.88) were independently associated with anti-HIV positivity, and the monophyletic clustering of HIV sequences from 14 cases indicated a common ancestry (21). Anti-HCV antibodies were present in high proportion among study participants, especially in those with HIV infection (21).

In Roka, Battambang province, Cambodia, an HIV outbreak involving 242 individuals in 2014–2015 was linked to injections received from an unlicensed health practitioner; 78% of the people infected with HIV were found to be HCV co-infected (22).

At a hospital-based pediatric antiretroviral clinic in Cape Town, South Africa, children with suspected horizontally acquired HIV infection were found through retrospective folder review of 2004–2014 patients and prospective interview and examination of patients from 2009 onwards (23). Thirty-two children acquired HIV, with transmission considered healthcare-associated in 15 (46.9%), community-associated in 10 (31.3%), possibly healthcare or community-associated in five (15.6 %); and unknown in two (6.3%) (23). Numerous studies, summarized by Gisselquist (24) and done previously in other (including sub-Saharan African) countries, demonstrated an even more important role for healthcare-associated HIV infections in children.

In 2019, an HIV outbreak occurred in Larkana District, Sindh, Pakistan. By December of that year, 881 (4.0%) of 21,962 children screened for HIV had tested positive. In an individually matched case-control study involving 406 cases aged < 16 years with HIV and 406 controls, the prevalence of anti-HCV antibodies was 6.5% (95% CI: 4.3–9.4) among cases and 1.0% (0.3–2.5) among controls (25). HIV infection was associated with a history of more injections and infusions (AOR 1.63; 95% CI: 1.30–2.04, *p* < 0.0001), blood transfusion (AOR 336.75; CI 23.69–4,787.01, *p* < 0.0001), surgery (AOR 399.75, CI 13.99–11,419.39, *p* = 0.0005), and increased frequency of private clinic (*p* < 0.0001) and government hospital visits (*p* < 0.0001) (25).

Unfortunately, over 5 years later, undiagnosed HIV infections persist in the community in Larkana, being common among children in Ratodero, where the outbreak was first noticed, and among both adults and children in surrounding areas (26). Exposure to unsafe injections (60%), unsafe blood transfusion (10%), and dental or surgical procedures (5%) are the most common modes of transmission (26).

An HIV cluster occurred in 2018–2023 among former spa clients of an unlicensed spa providing cosmetic injection services in New Mexico, USA, and was associated with receipt of platelet-rich plasma microneedling facials (27).

## Prevention of HIV and HCV parenteral transmission

It has long been well known how to prevent bloodborne infections (28). Sterility of needles and other equipment used in medical settings (including by dentists and in hemodialysis centers) and in rituals must be constantly controlled. Availability of auto-disabled syringes and needles, routine compliance with infection control precautions and efficient sterilization of medical instruments, including endoscopes, appropriate community information and enforcement of regulatory practices are extremely important. All blood donations must be adequately screened. Unnecessary medical injections, unnecessary transfusions, reuse of needles and syringes must be banned. Policies must be developed to enforce the use of sterilized equipment for scarifications and tattooing. Screening and eventual treatment of people in prison and of intravenous drug users should be implemented as well as infection control training programs and surveillance systems for nosocomial infections.

## Conclusions

It is essential to reduce parenteral transmission of HIV and HCV.

A targeted approach using combined testing for bloodborne viral infections could facilitate an early diagnosis in the general population. Unfortunately, a recent, comprehensive systematic review and meta-analysis examining the diagnostic performance of published predictive models for these infections in a wide range of populations and settings identified very large variations and a high risk of bias (29). Hence, efforts must be made to develop and evaluate more valid models.

In healthcare settings, it must be remembered that guidelines alone are insufficient for infection prevention in the absence of sufficient staffing. Healthcare workers are generally aware of guidelines for injection practices to prevent infection; however, they do not consistently follow them, due to high workload because of understaffing, or difficulty applying consistently aseptic technique (30). Higher workload among workers in larger hospitals can indeed lead to reduced compliance with guidelines in respect of medium-sized hospitals (30).

Finally, hospital-acquired infection underreporting must be reduced, developing a non-punitive reporting culture and favoring accurate, transparent, and constructive reporting (31).

## References

[1] Khumalo N Mankahla A Korsman S Gumedze F Basera W Ndyenga A . Scalp haircuts, keloids and blood-borne virus transmission risk in South Africa-The SHAKA study. PLoS ONE. (2025) 20:e0336213. doi: 10.1371/journal.pone.0336213 41259314 PMC12629497

[2] Sarkar S HIV: five children in India are infected from contaminated blood donations. BMJ. (2025) 391:r2310. doi: 10.1136/bmj.r2310 41184020

[3] Makuza JD Nisingizwe MP Rwema JOT Dushimiyimana D Habimana DS Umuraza S . Role of unsafe medical practices and sexual behaviours in the hepatitis B and C syndemic and HIV co-infection in Rwanda: a cross-sectional study. BMJ Open. (2020) 10:e036711. doi: 10.1136/bmjopen-2019-036711 32660951 PMC7359181

[4] Vento S Dzudzor B Cainelli F Tachi K. Liver cirrhosis in sub-Saharan Africa: neglected, yet important. Lancet Glob Health. (2018) 6:e1060–1. doi: 10.1016/S2214-109X(18)30344-9 30219314

[5] Venkatesh R Huang AS Gurmessa K Hsu EB. Understanding barriers to hepatitis C antiviral treatment in low-middle-income countries. Healthcare. (2024) 13:43. healthcare13010043 doi: 10.3390/healthcare13010043 39791650 PMC11720484

[6] Habib M Mohamed MK Abdel-Aziz F Magder LS Abdel-Hamid M Gamil F . Hepatitis C virus infection in a community in the Nile Delta: risk factors for seropositivity. Hepatology. (2001) 33:248–53. doi: 10.1053/jhep.2001.20797 11124843

[7] Guadagnino V Stroffolini T Rapicetta M Costantino A Kondili LA Menniti-Ippolito F . Prevalence, risk factors, and genotype distribution of hepatitis C virus infection in the general population: a community-based survey in southern Italy. Hepatology. (1997) 26:1006–11. doi: 10.1002/hep.510260431 9328327

[8] Bari A Akhtar S Rahbar MH Luby SP. Risk factors for hepatitis C virus infection in male adults in Rawalpindi-Islamabad, Pakistan. Trop Med Int Health. (2001) 6:732–8. doi: 10.1046/j.1365-3156.2001.00779.x 11555441

[9] el-Sadawy M Ragab H el-Toukhy H el-Mor Ael-L Mangoud AM Eissa MH . Hepatitis C virus infection at Sharkia Governorate, Egypt: seroprevalence and associated risk factors. J Egypt Soc Parasitol. (2004) 34:367–84. 15124747

[10] Idrees M Riazuddin S. Frequency distribution of hepatitis C virus genotypes in different geographical regions of Pakistan and their possible routes of transmission. BMC Infect Dis. (2008) 8:69. doi: 10.1186/1471-2334-8-69 18498666 PMC2409346

[11] Khattak MN Akhtar S Mahmud S Roshan TM. Factors influencing hepatitis C virus Sero-prevalence among blood donors in North West Pakistan. J Public Health Policy. (2008) 29:207–25. doi: 10.1057/jphp.2008.7 18523475

[12] Chung YS Choi JY Han MG Park KR Park SJ Lee H . A large healthcare-associated outbreak of hepatitis C virus genotype 1a in a clinic in Korea. J Clin Virol. (2018) 106:53–7. doi: 10.1016/j.jcv.2018.07.006 30075460

[13] Wu PY Hung MN Huang WL Yang JY Su CP. Hepatitis C outbreak in a respiratory care ward associated with frequent injections: Taiwan, 2017. J Microbiol Immunol Infect. (2021) 54:893–900. doi: 10.1016/j.jmii.2020.12.001 33342703

[14] Caminada S Mele A Ferrigno L Alfonsi V Crateri S Iantosca G . Risk of parenterally transmitted hepatitis following exposure to invasive procedures in Italy: SEIEVA surveillance 2000-2021. J Hepatol. (2023) 79:61–8. doi: 10.1016/j.jhep.2023.03.002 36935022

[15] Lim SH Lee S Lee YB Lee CH Lee JW Lee SH . Increased prevalence of transfusion-transmitted diseases among people with tattoos: a systematic review and meta-analysis. PLoS ONE. (2022) 17:e0262990. doi: 10.1371/journal.pone.0262990 35085358 PMC8794209

[16] Foerster M Zins M Goldberg M Ribet C Kab S McCarty R . Tattoo practices and risk of hepatitis B and hepatitis C infection in the French Constances study. Int J Infect Dis. (2026) 163:108288. doi: 10.1016/j.ijid.2025.108288 41386394 PMC12830372

[17] Kassa GM Lim AG Tamiru MT Alamneh TS Vickerman P Dagne E . DESTINE NIHR Global Health Research Group. Risk factors for hepatitis C virus among the general population in sub-Saharan Africa – an analysis of systematic review data. J Viral Hepat. (2025) 32:e70065. doi: 10.1111/jvh.70065 40888503 PMC12400790

[18] Singh J Stoitsova S Zakrzewska K Henszel L Rosińska M Duffell E. Healthcare-associated hepatitis B and C transmission to patients in the EU/EEA and UK: a systematic review of reported outbreaks between 2006 and 2021. BMC Public Health. (2022) 22:2260. doi: 10.1186/s12889-022-14726-0 36463162 PMC9719626

[19] Brewer DD Potterat JJ Roberts JM Jr Brody S. Male and female circumcision associated with prevalent HIV infection in virgins and adolescents in Kenya, Lesotho, and Tanzania. Ann Epidemiol. (2007) 17:217–26. doi: 10.1016/j.annepidem.2006.10.010 17320788

[20] Asa GA Fauk NK Ward PR. Traditional male circumcision and the risk for HIV transmission among men: a systematic review. BMJ Open. (2023) 13:e072118. doi: 10.1136/bmjopen-2023-072118 37208134 PMC10201269

[21] Patil S Rao A Pathak P Kurle S Mane A Nirmalkar A . Unsterile injection equipment associated with HIV outbreak and an extremely high prevalence of HCV—A case-control investigation from Unnao, India. PLoS ONE. (2020) 15:e0243534. doi: 10.1371/journal.pone.0243534 33275646 PMC7717531

[22] Rouet F Nouhin J Zheng D Roche B Black A Prak S . Massive iatrogenic outbreak of human immunodeficiency virus type 1 in rural Cambodia, 2014–2015. Clin Infect Dis. (2018) 66:1733–41. doi: 10.1093/cid/cix1071 29211835 PMC5963970

[23] Myburgh D Rabie H Slogrove AL Edson C Cotton MF Dramowski A. Horizontal HIV transmission to children of HIV-uninfected mothers: a case series and review of the global literature. Int J Infect Dis. (2020) 98:315–20. doi: 10.1016/j.ijid.2020.06.081 32615324

[24] Gisselquist D. Comprehensive investigations of unexplained HIV infections to protect children. Int J Infect Dis. (2020) 101:58. doi: 10.1016/j.ijid.2020.09.1462 33002617

[25] Mir F Nathwani AA Simms V Abidi SH Siddiqui AR Hotwani A . Factors associated with HIV infection among children in Larkana District, Pakistan: a matched case-control study. Lancet HIV. (2021) 8:e342–52. doi: 10.1016/S2352-3018(21)00049-7 34087096

[26] Jamil MS Pasha MS Ali A Memon S Soomro AA Dharejo Z . Identifying undiagnosed HIV infected individuals in a community-based door to door testing initiative in outbreak affected areas of Larkana and surrounding districts, Pakistan. Lancet Reg Health Southeast Asia. (2026) 44:100713. doi: 10.1016/j.lansea.2025.100713 41551377 PMC12810572

[27] Stadelman-Behar AM Gehre MN Atallah L Clarke T Leonso AA Jojola F . Investigation of presumptive HIV transmission associated with receipt of platelet-rich plasma microneedling facials at a spa among former spa clients – New Mexico, 2018-2023. MMWR Morb Mortal Wkly Rep. (2024) 73:372–6. doi: 10.15585/mmwr.mm7316a3 38662678 PMC11065465

[28] Centers for Disease Control (CDC). Update: universal precautions for prevention of transmission of human immunodeficiency virus, hepatitis B virus, and other bloodborne pathogens in health-care settings. MMWR Morb Mortal Wkly Rep. (1988) 37:377–82, 387–8. 2836717

[29] Leber W Farooq HZ Panovska-Griffiths J Larvin H Baggaley RF Divall P . Risk prediction models for targeted testing of HIV, hepatitis B and hepatitis C: a systematic review and meta-analysis. BMC Infect Dis. (2025) 25:1648. doi: 10.1186/s12879-025-11921-3 41291465 PMC12648812

[30] Park JW Park S Lee E Kim T Kim ES Kim B . Latent factors affecting safer injection practices that can reduce infections and how education can improve th‘em. PLoS ONE. (2024) 19:e0308567. doi: 10.1371/journal.pone.0308567 39423176 PMC11488737

[31] Luo Y. Hospital infection underreporting rate: an analysis of causes and research on improvement measures. Soc Med Health Manag. (2023) 4:55–60. doi: 10.23977/socmhm.2023.040408

